# The transiting dust clumps in the evolved disc of the Sun-like UXor RZ Psc

**DOI:** 10.1098/rsos.160652

**Published:** 2017-01-25

**Authors:** Grant M. Kennedy, Matthew A. Kenworthy, Joshua Pepper, Joseph E. Rodriguez, Robert J. Siverd, Keivan G. Stassun, Mark C. Wyatt

**Affiliations:** 1 Institute of Astronomy, University of Cambridge, Madingley Road, Cambridge CB3 0HA, UK; 2 Leiden Observatory, Leiden University, PO Box 9513, 2300 RA Leiden, The Netherlands; 3 Department of Physics, Lehigh University, 16 Memorial Drive East, Bethlehem, PA 18015, USA; 4 Harvard-Smithsonian Center for Astrophysics, 60 Garden Street MS-78, Cambridge, MA 02138, USA; 5 Department of Physics and Astronomy, Vanderbilt University, 6301 Stevenson Center, Nashville, TN 37235, USA; 6 Las Cumbres Observatory Global Telescope Network, 6740 Cortona Drive, Suite 102, Santa Barbara, CA 93117, USA; 7 Department of Physics, Fisk University, 1000 17th Avenue North, Nashville, TN 37208, USA

**Keywords:** variable stars, protoplanetary discs, debris discs, circumstellar matter

## Abstract

RZ Psc is a young Sun-like star, long associated with the UXor class of variable stars, which is partially or wholly dimmed by dust clumps several times each year. The system has a bright and variable infrared excess, which has been interpreted as evidence that the dimming events are the passage of asteroidal fragments in front of the host star. Here, we present a decade of optical photometry of RZ Psc and take a critical look at the asteroid belt interpretation. We show that the distribution of light curve gradients is non-uniform for deep events, which we interpret as possible evidence for an asteroidal fragment-like clump structure. However, the clumps are very likely seen above a high optical depth midplane, so the disc’s bulk clumpiness is not revealed. While circumstantial evidence suggests an asteroid belt is more plausible than a gas-rich transition disc, the evolutionary status remains uncertain. We suggest that the rarity of Sun-like stars showing disc-related variability may arise because (i) any accretion streams are transparent and/or (ii) turbulence above the inner rim is normally shadowed by a flared outer disc.

## 1. Introduction

Discs of gas and dust surround essentially all young analogues of our Sun [1]. The lifetime of the gas in these discs is very short compared with the stellar lifetime, and within a few million years has accreted onto the star, been lost to space in photoevaporative flows, and contributed to building planets. The evolution of the dust during this phase is uncertain, but the existence of gas giant planets makes it clear that planetary building blocks, and of course some planets, form on a similar or shorter timescale.

Beyond the first few million years, a typical star hosts a planetary system, the components being the planets themselves and a residual disc of small bodies. These ‘planetesimals’—the asteroids and comets—make up the ‘debris disc’, where the standard picture is that destructive collisions between them generate a size distribution of fragments that extends down to micrometre-sized dust [2–4].

The state of planetary systems as they emerge from the gas-rich phase is uncertain. Planets’ locations are not finalized at this epoch, but may move by interacting with other stars, planets and/or planetesimals in the system [5–7]. Similarly, the state and origin of the debris disc is uncertain. At stellocentric distances near 1 AU, the region of interest in this article, it could be that dust observed at this time is related to the final stages of planet formation [8,9], originates in young analogues of our asteroid belt [10], is a signature of comets scattered inwards from more distant regions [11] or is simply a remnant of the gas-rich disc that has yet to be dispersed [12]. In the absence of gas detections that argue for the latter scenario, discerning among these various scenarios, which are not mutually exclusive, is difficult.

A promising way to probe these inner regions is by observing temporal variability [13]. Optical and IR stellar variation has been studied for decades [14,15], and has recently been reinvigorated by large-scale efforts [16,17] and as a side effect of large-scale surveys for transiting planets [18,19]. Of many different classes of variables, those of most interest and relevance here are the ‘UXors’, named for the prototypical system UX Orionis [15]. These are usually Herbig Ae and late-type Herbig Be stars [20], and typically show several magnitudes of extinction that is generally attributed to variable obscuration by circumstellar dust [15,21,22].^1^ Three related arguments that favour circumstellar dust as the cause are (i) a maximum depth of dimming events of roughly three magnitudes, suggesting that a few per cent of the visible flux is not directly from the star, but scattered off a disc that surrounds the star and remains visible even when the star itself is completely occulted, (ii) ‘blueing’, where the star is reddened for small (≲1 mag) levels of dimming but returns to the stellar colour (i.e. becomes ‘bluer’) for the very deep (≳1 mag) events where the star is mostly occulted—the reddening indicates dimming by circumstellar dust, and a stellar colour is typical of light scattered off circumstellar dust [28], and (iii) increased polarization fraction during dimming events, caused by a greater fraction of the flux being contributed by dust-scattered light (e.g. [29], which also shows that the surrounding dust does not reside in a spherical shell). UXors therefore reveal information on the degree of non-axisymmetry, the ‘clumpiness’, of dust orbiting a star on spatial scales similar to the star itself. The observations can span multiple orbits to test for repeated dimming events [30], and by using different bandpasses and polarization can estimate dust grain sizes [28,31].

In the majority of UXor-like cases (i.e. those related to obscuration by dust), including other classes such as ‘dippers’ [17,32], the processes causing young stars to vary are attributed to gas-rich protoplanetary discs. For Herbig Ae/Be stars, the obscuration is thought to be caused by hydrodynamic turbulence that lifts dust above the puffed up inner rim of a self-shadowed disc [33]. For the dippers, which are observed around low-mass stars, the obscuration is attributed to dust in accretion streams that link the inner disc and the stellar surface, and/or to variations in the height of the inner disc edge [30,32,34]. The common theme is therefore that the location of the occulting dust is as close to the star as physically possible, being set by sublimation [35]. These systems tell us about the nature of turbulence and accretion in gas-dominated discs, but so far reveal little about how these discs transition to the debris phase and the subsequent evolution.

Here, we focus on RZ Psc, a star that shows UXor-like variability [36–38]. As a young K0V-type star with no evidence for gas accretion and a strong infrared (IR) excess, this system appears unique among UXors and may provide us new information on the structure of inner planetary systems during or following dispersal of the gas disc [39,40]. However, the IR excess indicates the over 5% of the starlight is intercepted by the disc, which is a level more akin to gas-rich protoplanetary and transition discs than debris discs. Specifically, we use a decade of ground-based optical photometry of RZ Psc (§3) to draw conclusions on dust location (§4), and discuss the possible disc structure and evolutionary state in §5. We conclude in §6.

## 2. A clumpy dust ring near 0.5 AU?

There is significant evidence that the optical variations seen towards RZ Psc are caused by circumstellar dust: (i) during dimming events, the colour becomes redder [36,41] in a way consistent with that expected for dust [42,43], (ii) the maximum depth is about 2.5 mag and during these events, the colour returns to near stellar values, suggesting that the remaining emission is from light scattered off the circumstellar dust (i.e. the star is fully occulted [28,42], and (iii) the polarization fraction increases during the transits, as expected if an increasing fraction of the light is scattered off a disc of circumstellar dust [28,38,44].

What separates RZ Psc from other UXors (and dippers) is (i) the spectral type is K0V rather than Herbig Ae/Be for UXors and late K to M type for dippers, (ii) the occulting dust lies well beyond the sublimation radius, and (iii) the star is not associated with a star-forming region so is inferred to be a few tens of millions of years old. The dust distance has been inferred from the speed of ingress of dimming events, which was previously estimated as about 0.6 AU (for circular orbits [40]). Corroborating evidence comes from approximately 500 K temperature of the dust seen in the mid-IR, which places it near 0.4–0.7 AU (depending on optical depth) and therefore at a location consistent with the occulting dust [40]. The distance to RZ Psc is unknown, but as an apparently isolated star that shows Li absorption the age has been estimated as a few tens of Myr, and therefore beyond the age at which a gas-rich disc would normally exist [39,45]. Further distinguishing features are that the duration of the dimming events is consistently short compared with other UXors, a few days rather than days to a few weeks, and that no near-IR (i.e. K-band) excess or accretion signatures are seen [45], so interpretations related to accretion of disc material onto the star [21,30,34] are unlikely.

Thus, the potentially compelling and unique aspect for RZ Psc is that we are observing dimming events from dust in a main-sequence planetary system that resides at about 0.5 AU. This dust is also seen in thermal emission, so deriving joint constraints on the dust properties and structure may be possible. As argued by de Wit *et al.* [40], a picture is emerging in which RZ Psc is surrounded by a massive young version of our own asteroid belt, in which planetesimals are continually being destroyed. These collisions generate the large collective surface area of small dust that emits strongly in the mid-IR, and the system geometry means that this dust also sometimes passes in front of the star.

While this asteroid belt picture is intriguing, and makes RZ Psc a system that could be of great interest and worthy of detailed study, it is not the only possibility. Well over 1% of the starlight is reprocessed by the circumstellar disc, which is more typical of the primordial gas-rich discs seen around nearly all young stars. The discovery of systems such as HD 21997, that appear to be a few tens of Myr old and host gas-rich discs [46], shows that stellar age is not a perfect indicator of disc status. Thus, a considerable part of our analysis focuses on the question of the status of the disc around RZ Psc.

Given the proposed interpretation related to individual planetesimal disruptions, rather than hydrodynamics, it is perhaps surprising that to date the dimming events are not seen to be periodic [40,47]. The only cyclical variation seen in light curves for RZ Psc is a 12.4 year variation with an amplitude of 0.5 mag, which is attributed to either a magnetic cycle or precession of an otherwise unseen outer disc owing to perturbations from an unseen companion [40].

## 3. Time series photometry

### 3.1. Optical

To study the temporal variability of RZ Psc, we use two seasons of public data from the wide-angle search for planets (WASP [48,49]), and nine seasons of data from the Kilodegree Extremely Little Telescope North (KELT-North [50]). We also collected, but ultimately did not use, photometric observations of RZ Psc from a wide variety of other sources ([15,36,41,44,51–53], the Catalina Sky Survey, the American Association of Variable Star Observers, the All-Sky Automated Survey). Aside from the Harvard plate photometry published by Gürtler *et al.* [37], we have not sought unpublished photometry, so the light curve remains incomplete.^2^

Here, we focus on the WASP and KELT-North data, as it has not been previously analysed and has considerably higher cadence (many measurements per night) and temporal coverage (nightly, weather permitting) than other datasets. The WASP data from 2004 and 2006 are public and were obtained from an online archive.^3^ These data were processed in a manner similar to that described by van Werkhoven *et al*. [54], where common-mode variations were removed using 50 quiet nearby stars. The WASP bandpass is broad, with roughly uniform transmission from 400 to 700 nm [48]. The KELT-North data, 2006–2014, were used in raw form, the only specific treatment being a 4% relative correction being made to ensure observations taken in the ‘east’ and ‘west’ telescope orientations have the same calibration. The bandpass is redder than for WASP, with most transmission between 500 and 800 nm [50]. For a full description of the KELT-North data reduction, see [55].

**Figure 1.**
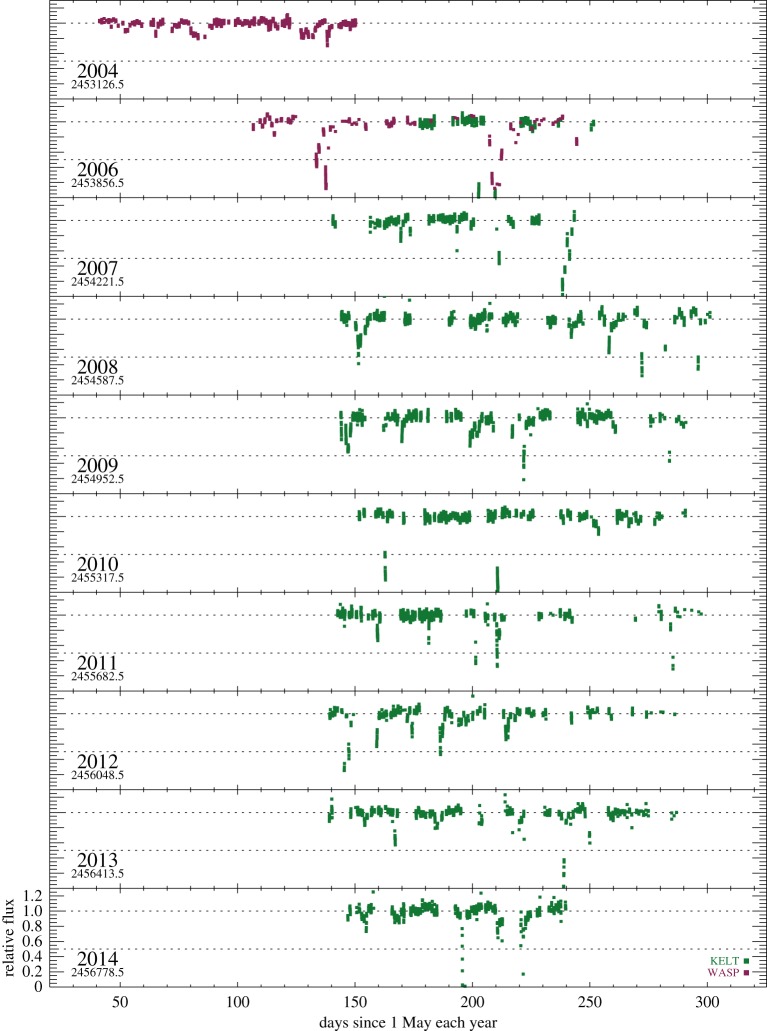
WASP and KELT-North data. Photometry is shown in dimensionless form, relative to a quiescent level of 1, and was converted from observed magnitudes as described in the text.

We normalized each year’s data from each instrument separately by converting magnitudes to flux density and dividing out the sigma-clipped median, so the light curve has an out-of-occultation baseline of 1. In doing so, we are assuming that variations owing to the slightly different filter bandpasses are unimportant. Each row in figure 1 shows a season’s data, starting on 1 May each year (JD also indicated). Most year’s data therefore extend into the next year, so the ‘2006 data’ refers to data from the 2006/2007 observing season.

#### 3.1.1. Qualitative light curve overview

It is clear from figure 1 that RZ Psc undergoes the very deep dimming events that are typical of UXors. These are seen a few times each observing season and vary in complexity, with a few extended events (e.g. 2006) and a greater number of ‘neater’ single events (e.g. 2010). In some years, there is also significant variability at shallower depths. Of particular note is the pair of deep events in 2006; these appear to be about 70 days apart, and given the suggestion that the putative asteroid belt analogue resides near 0.5 AU a natural inference is that these two events are related. If true, this repetition corresponds to a semi-major axis of about 0.3 AU, which given uncertainties in the true disc spectrum could be consistent with the location of the asteroid belt. In 2004, there are about 100 days of near-consecutive nights of data and no deep events, so either the true period is longer than 100 days or dust clumps can be created (and perhaps destroyed) on timescales of a year or so.

**Figure 2.**
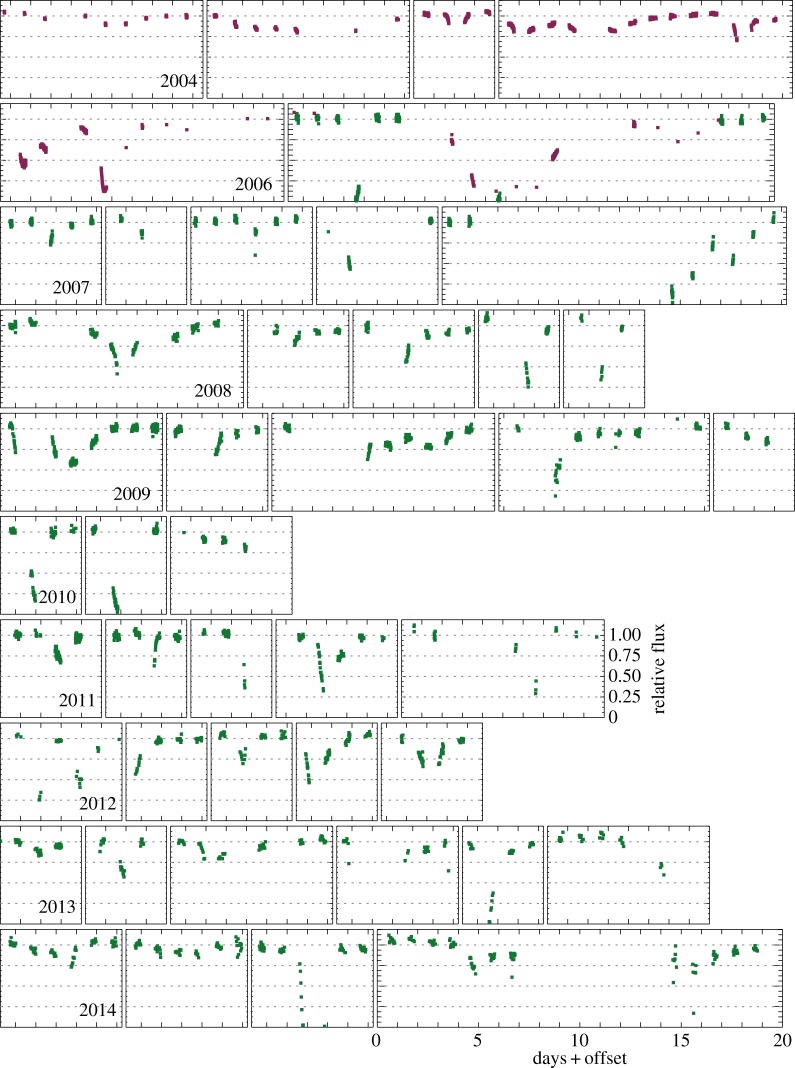
WASP and KELT-North data, focusing on dimming events. The vertical and horizontal scales in each subpanel are the same.

In figure 2, we have selected most of the events from each year and shown them at a greater temporal resolution. The scale in each panel is the same, so wider boxes simply cover longer events. Most events appear to last at least a few days, suggesting that only having nightly coverage does not seriously hinder our ability to detect most events. However, the events are sufficiently short and irregular that the true shape of events remains uncertain. While it is likely that interpolation of the photometry for the fourth event in 2011 (i.e. the fourth box from the left in the row corresponding to 2011 in figure 2) would resemble the true light curve, this assumption seems very unlikely to yield the true evolution of more complex events like those in 2006.

Nevertheless, figure 2 shows an unprecedented view of dimming events seen towards RZ Psc, and that key information on the ingress and egress of dimming events is present. The dimming rate is such that it can be resolved temporally, and hence the velocity and radial location of the dust clumps estimated. While such estimates have been made in the past based on one or two individual events [36], these data make them possible for an ensemble of tens of events.

A fairly basic question is whether the light curve could result from objects that all have the same properties or whether a range is required. Given the existence of both long shallow events and short deep events, at a minimum, the clumps must vary in size and/or velocity across the face of the star, but probably also have different optical depths. The star can be completely occulted, so the clumps can be optically thick and star-sized, but it has already been shown that the events are not grey, so the clumps must have a density gradient rather than have sharp edges. Where sufficient data exist, it is clear that not all events have the same relative shape, so the shape of the clumps varies. Thus, the broad picture is of roughly star-sized clumps, whose shape and orbital elements vary. The fact that the dimming events can be shown in a series of panels with the same scale in figure 2 suggests that the range over which these properties vary is of order factors of a few, not many orders of magnitude.

### 3.2. Infrared

While the optical photometry reveals information about how RZ Psc is itself dimmed, IR photometry beyond a few micrometres is dominated by emission from the circumstellar disc. Thus, IR variation reveals information about how the emitting surface area, temperature and perhaps composition, of the dust change with time. Such variation is indeed apparent, both from comparison of an AKARI 18 μm non-detection at a lower level than the WISE 22 μm detection, and from several individual WISE measurements taken at six month intervals.

Motivated by this variation, we obtained VLT/VISIR observations of RZ Psc; an N-band (10 μm) spectrum on 16 August 2016 and Q-band (18μm) photometry on 27 July 2016 (programme 097.C-0217). These data, and the related calibration observations, were reduced using the standard ESO esorex pipeline. The wall-clock integration time for the spectrum was 50 min at an airmass of 1.66, and was calibrated using an observation of HD 189831 taken immediately afterwards at an airmass of 1.63. The spectra in individual chop/nod cycles are consistent, so the shape of the spectrum is reliable. The absolute calibration is uncertain at approximately 10% levels [56], which is sufficiently precise for our purposes here. The spectrum was trimmed to mask highly uncertain regions shortward of 8 μm, and longward of 13 μm, and near the telluric absorption at 9.5 μm. The Q-band photometry took 45 min at an airmass of 1.65–1.7, and was calibrated against an observation of HD 2436 taken immediately afterwards at an airmass of 1.5. Photometry of RZ Psc and HD 2436 was done using a 0.9^′′^ radius aperture and a sky annulus from 1 to 2^′′^. In addition to the conversion from adu/s to Jansky using HD 2436, we applied an additional upward correction to account for the slightly lower airmass for the calibrator (exp⁡(0.3[1.675−1.5]≈1.05, where an extinction of 0.3 per unit airmass was used, derived from archival calibration data using a method similar to that of Verhoeff *et al.* [57]). Uncertainties were estimated as the standard deviation of the flux density in 50 apertures around RZ Psc. The final calibrated flux is 86±10 mJy.

**Figure 3.**
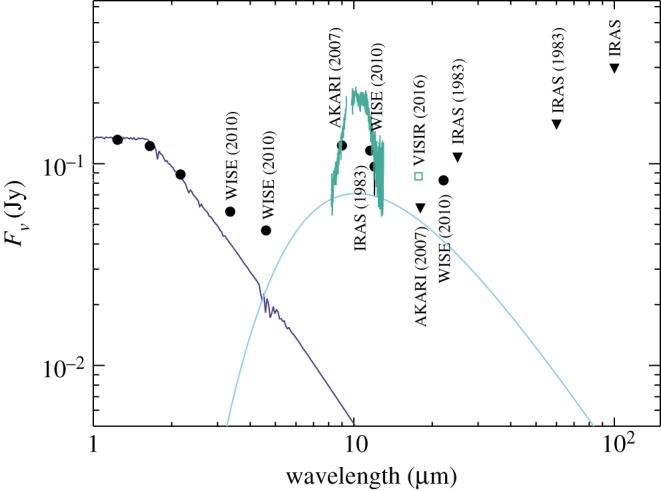
Flux density distribution of RZ Psc, including 2MASS, WISE, AKARI, VISIR and IRAS data and their (approximate) year of observation. The dark blue line shows a stellar photosphere model at the approximate stellar temperature of 5350 K, and the light blue line a 500 K blackbody. The latter is not a fit, but an approximate continuum level that illustrates that the WISE 3.4 and 22 μm photometry cannot both be accounted for with a single blackbody, if the silicate feature seen with VISIR was present in 2010.

The VISIR spectrum and photometry are shown in figure 3, which also shows the other available near- to far-IR photometry. The absolute level of the spectrum agrees well with the IRAS, WISE (from the ALLWISE catalogue) and AKARI observations; that four observations spanning over 30 years are consistent suggests that while this part of the spectrum may vary, the shape and level shown is typical. The same cannot be said near 18 μm, where the AKARI and VISIR (and perhaps WISE depending on the disc spectrum) flux densities are inconsistent. The flux near 10 μm being relatively constant, and the 18μm flux changing could be indicative of shadowing of an outer disc by an inner disc; that is, evidence that the disc around RZ Psc has significant radial extent. This type of behaviour is seen as ‘see-saw’ variability in IR spectra towards some transition discs [58,59].

The spectrum clearly shows solid-state emission (and the continuum level is not actually clear), which indicates (i) that the dust is of order micrometres in size and (ii) that the dust includes silicates. The smooth rise and fall suggests that the silicates are largely amorphous; crystalline silicates have sharper features, notably a depression between two peaks at 10 and 11 μm, rather than the flat top seen here. Other systems thought to host bright asteroid belt analogues (rather than gas-rich discs), such as HD 69830, BD+20 307 and HD 113766A, tend to show crystalline features [60–62], which may argue against such a scenario for RZ Psc. However, such comparisons are largely speculative as there is also a high degree of variation among silicate features, for both gas-rich and gas-poor discs.

**Figure 4.**
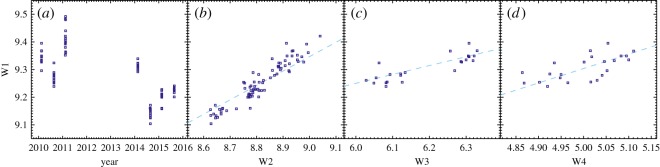
WISE epoch photometry at 3.4, 4.6, 12 and 22 μm (W1, W2, W3 and W4, in magnitudes). Panel (*a*) shows the time variation in W1 over 5.5 years. Panels (*b*–*d*) show how W1 correlates with W2, W3 and W4, which do not have observations at all W1 epochs. The dashed lines show the slope expected for constant disc flux variation with wavelength (the variation is smaller in W1/2, because the total flux is not dominated by the disc).

To further explore this variability, we use the WISE ‘epoch’ photometry, for which the telescope scanning strategy results in clusters of measurements that are spaced six months apart. These data appear at approximately days 70 and 250 in the relevant years in figure 1, but do not coincide with any optical dimming events. Photometry is not available in all four channels since launch in early 2010, owing to exhaustion of the coolant after 7.7 months and a 2.5-year hiatus from mid-2011 to 2014 (see [63,64]). These data are shown in figure 4, where figure 4*a* shows the 3.4 μm magnitude as a function of time, and that there is significant variation on six month timescales, and an even greater variation overall. Inspection of the individual clusters, which are on hour-to-day timescales, shows no significant variation with time. The dashed lines have the slope expected for disc brightness variation that is independent of wavelength; the slopes are not exactly 1, because the total flux is not dominated by the disc near 3–5 μm, and hence the slopes are slightly flatter. Comparing the observed and expected correlations, we conclude that the data do not show significant evidence for changes in the spectral shape (i.e. changing temperature or composition). However, the ratios including 12 and 22 μm observations are most sensitive to these changes, but only include the first two sets of observations where the brightness changes were relatively small.

Nevertheless, the amplitude of the change in 3.4 μm brightness over 5 years is about 30%. Considering that the disc flux density is only 45% of the total flux at this wavelength, the disc brightness increased from 2010 to 2015 by about a factor of two. A similar variation can be inferred by comparing the 18 μm upper limit from AKARI in 2007 and our VISIR measurement in 2016. Given these increases, it is surprising that the N-band spectrum does not appear much higher than the IRAS, AKARI and WISE photometry. A possible explanation would be that the increased emission originates in larger grains, which would result in greater continuum flux but similar levels in spectral features. However, without wider spectral and more frequent temporal coverage quantifying such effects is difficult. This level of IR variation is seen towards both protoplanetary [58,65] and debris discs [13,66,67], so these data provide little means to distinguish between scenarios.

## 4. Where are the occulting bodies?

The main part of our analysis concerns attempts to extract information from the optical light curve, taking advantage of the great number of dimming events seen over 10 seasons. In this section, we focus on the radial location of the bodies (‘clumps’) that pass in front of RZ Psc, first searching for periodicity associated with repeat events, and then using the light curve gradients to constrain the projected velocities. This analysis primarily focuses on what can be gleaned from the light curves, and the implications of these results for different clump origins are then explored in §5.

### 4.1. Search for periodic dimming events

We begin by estimating the lifetime of an occulting clump as a check on the plausibility that dimming events should repeat. The angular rate at which clumps are sheared out is *R*d*Ω*/d*R*=−3*Ω*/2. Accounting for shear in forward (interior) and backward (exterior) directions, the shear velocity across a clump of radius *R*_cl_ is then

4.1 $$v_{\mathrm{sh}}=3R_{\mathrm{cl}}\Omega ,$$

so the clump expansion rate owing to shear in units of clump radii is only three times the orbital frequency. That is, after one orbit a clump will be stretched by a factor of 6*π*, and the radial and vertical optical depth will be roughly 6*π* lower (though it might still be optically thick). Thus, clumps that are not bound by their own self-gravity are expected to have a short lifetime at optical depths that are large enough to cause detectable dimming events, but could cause repeated dimming events if they are initially optically thick.

The temporal coverage of the observations in an individual season is 100–150 days. Thus, if the occulting material resides in an asteroid belt closer than approximately 0.5 AU, periodicity in the dimming events may be visible in a single season’s data. Longer orbital periods may be visible across seasons, though the six month gap between seasons makes unambiguously linking events harder. Non-detection of periodicity would imply that the data are not sufficient or that strict periodicity does not exist. An intermediate possibility is that occultations happen with a range of periodicities, perhaps reflecting their origin in a radially broad region, and that discerning this scenario from randomly occurring occultations is not possible given the data.

In an attempt to find the expected periodicity, we tried several approaches. These are similar in that they aim to quantify whether some feature in the light curve is repeated again at a later time, but differ in how well they reveal evidence for a periodic signal. We found no evidence for events that are related from one year to another, so focus on statistics derived from individual seasons’ data (though these are sometimes combined).

#### 4.1.1 Autocorrelation

We first used autocorrelation to search for periodicity rather than used methods related to Fourier transforms (e.g. periodograms). The motivation being that an individual transit event may be followed by another some number of days later, and perhaps repeat a few times, but other similarly (but not exactly) separated events may happen years later or earlier with a phase that is totally different. We therefore used the discrete autocorrelation function (DACF) proposed by Edelson & Krolik [68], though do not include uncertainties on individual measurements. For a time series with measurements *a*_*i*_ at times *t*_*i*_, the DACF first computes the mean $\bar{a}$ from the light curve (here we used the sigma-clipped mean to remove the dimming events), then, for each pair of points *a*_*i*_, *a*_*j*_ (with *i*≠*j*) computes $U_{ij}=((a_{i}-\bar{a})(a_{j}-\bar{a})/\sigma_{a}$, with each *U*_*ij*_ associated with a time lag Δ*t*_*ij*_=*t*_*j*_−*t*_*i*_. A series of time lags centred at times *t*_lag_ with width Δ*t*_lag_ are then used as bins, and the average in each bin is the DACF. The DACF is not computed for lag bins with no data. The units of the DACF are standard deviations of the light curve *σ*_*a*_ (again calculated using sigma clipping).

**Figure 5.**
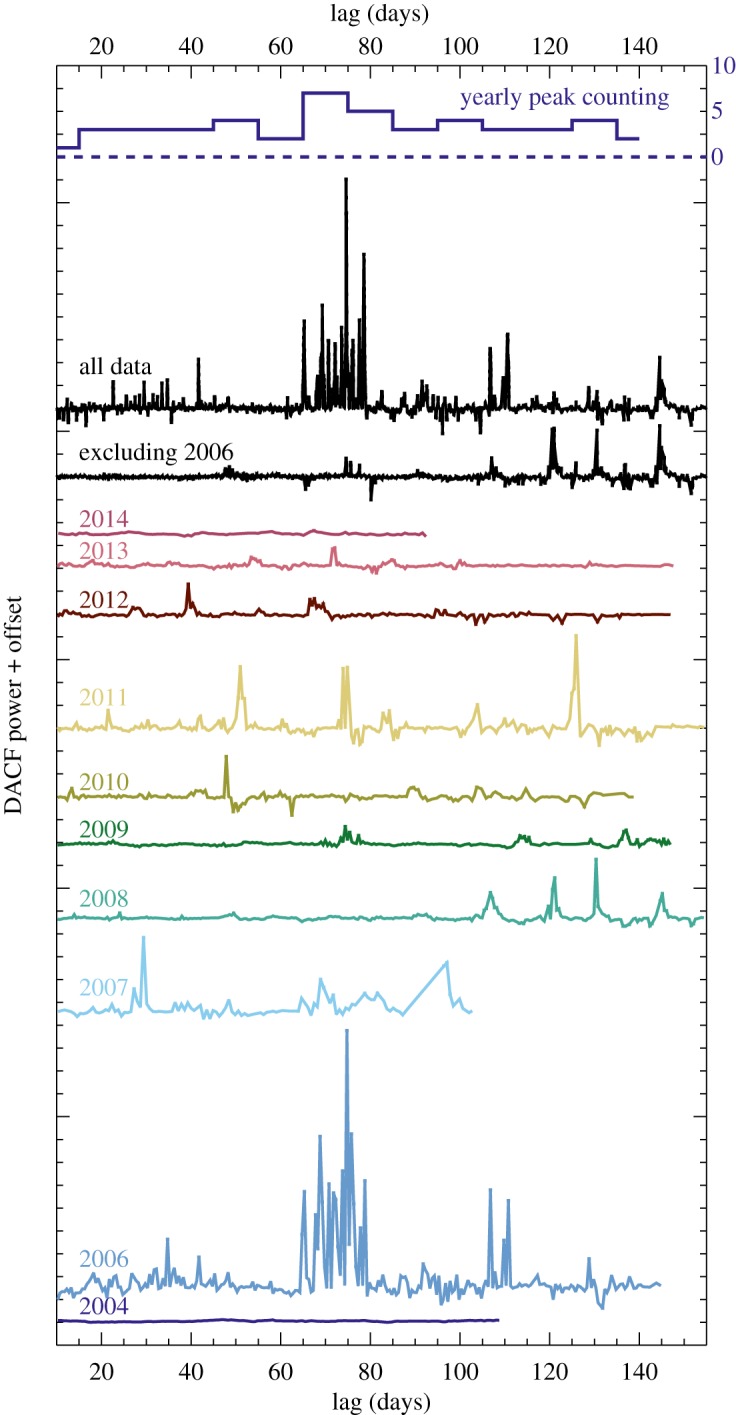
Discrete autocorrelation function for yearly WASP and KELT-North data, computed for lags between 10 and 155 days. The second and third lines from the top show all data, and 2006-excluded data. The topmost line shows the number of years that show a peak more than 3*σ* above the clipped DACF mean within each 5 day bin. The peak at 70 days is 7 years, the dashed line is zero and the *y*-axis scale is shown to the right.

The results are shown in figure 5 for time lags (i.e. trial periods) of 10–155 days in half-day bins. Comparison of these with the light curves shows that the DACF recovers most, but not all, events. Conversely, not all DACF peaks are necessarily associated with real repeat events, as there may of course be multiple distinct clumps orbiting the star at any given time. Not all pairs of events show a strong DACF signal, as they can comprise only a few measurements and the mean for that *t*_lag_ dominated instead by a much larger number of measurements elsewhere in the light curve closer to the quiescent level (i.e. near $\bar{a}$). Our attempts to avoid this issue by using autocorrelation on interpolated data yielded mixed results; heavy filtering, such as setting all data above a given level to 1, was needed for results similar to the DACF shown in figure 5.

While several strong peaks appear in the DACF of all data, most of these arise from 2006, as can be seen in the DACF when these data are excluded. Some peaks remain near 70 days, as well as at 120 and 145 days, and the latter two could be aliases of periods near 60–70 days, arising simply because an event was missed. That is, the irregular sampling means that absence of evidence of power at some period in the DACF is not evidence of absence.

The pair of events separated by 70 days in 2006 provides the strongest signal, and most other years also show events near this period (2007, 2009, 2011–2014). To illustrate these numbers the topmost line in figure 5 quantifies the number of years that show a peak, in 10 day bins. The peak of 7 years is at 65–75 days, which is suggestive but not conclusive because a K–S test shows that this distribution is consistent with being uniform in period.

#### 4.1.2. Iterative event finding

In an attempt to avoid some of the difficulties arising from the DACF, we tried a similar approach that first identifies individual occultation events and then computes the time delays between them. The main aim was to identify and use all events in a way that avoids biases related to the sampling of the data and the different relative depths of potentially repeated events. By repeating this prescription for synthetic data, we are able to test different scenarios for how the occultations do or do not repeat.

For this approach, an event is initially identified as the lowest point that is 6*σ* below the mean, where the mean and standard deviation are again estimated by sigma-clipping. This lowest point is noted, as are all immediately adjacent points that are also below the threshold noted. The points so included constitute a single dimming event. The points belonging to this event are removed from the light curve and the process repeated until no significant events remain. The time of the event is the time of the lowest point, and the duration the time between the two endpoints that are consistent with the quiescent level. Thus, if an event is in a region of sparse sampling, the duration can appear to be longer than it probably is, though we discard any events that occur at the beginning or end of an observing season to avoid unreasonably long events.

For a given set of event times and durations, the range of possible times between events is then calculated using the maximum allowed by the duration. This calculation is done for all combinations, yielding *N*(*N*−1)/2 inter-event time ranges. These ranges are then ‘stacked’ into a histogram (i.e. counting +1 for time differences within a given bin) that shows a measure of the power present at a given time difference.

**Figure 6.**
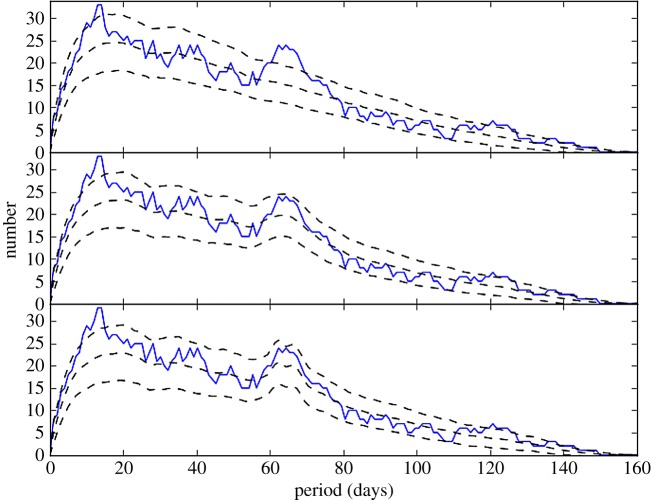
Power at a given period from the iterative event finding. The solid line shows the power from the data, and is the same in each panel. The dashed lines show the mean and ±1*σ* power from simulated dimming events, which from top to bottom are: random, periodic between 64 and 75 days with three repeats, and periodic at 64 days with 10 repeats.

The solid lines in figure 6 show this power in histogram form (the same in each panel), generated from 60 events that were identified in the light curve. The *y*-axis should be interpreted as the number of events that are consistent with that period in the *x*-axis. As before, there is evidence for a peak, now slightly shifted to near 65 days. In contrast to the DACF analysis, this power does not all arise from a single year. Most is contributed by 2004 and 2009, but exclusion of events from these years results in a similar (but noisier) histogram.

To test what could have been detected, and quantify the variation in power expected, we created synthetic light curves with the same temporal sampling as the WASP and KELT-North data. To do this, we set the flux to 1, and randomly injected a number of dimming events with a flux of 0.1. These events are therefore relatively easy for the algorithm to detect, but suffer the same sampling issues. We tried individual randomly occurring events, and periodic events that repeat a fixed number of times. All events have durations randomly distributed between 1 and 4 days, similar to the observed events. For random events, the remaining parameter in this model is simply the number of events—this is the total number over the 11 year period covered by the WASP and KELT-North data, including when measurements were not being taken. For repeating events, the range of periods and the number of repeats are additional parameters. To estimate the level of variation in the power spectrum, we repeated the process of injecting synthetic events 500 times, and in each bin estimate the mean and standard deviation of the power.

The results are shown in figure 6, where the dashed lines show the mean and ±1*σ* deviations from the simulations (and solid lines show the data). Each panel shows a different scenario, from top to bottom these are: 295 random events, 100 events with periods between 64 and 75 days and three repeats, and 100 events with a period of 64 days and three repeats. The purely random events are marginally disfavoured; while the data lie outside the dashed lines, these are only 1*σ*. Nevertheless, the middle and bottom panels show that the possible peak near 65 days could be caused by events with a range of periods, but that only a single period of 64 days is actually needed and yields a slightly stronger peak. The remaining excess periodicity near 10 days is not accounted for by any of the models, but could be an indication that on this timescale events are related (e.g. a clump that has separated into several). That the real signal nearly lies within the 1*σ* variation expected for randomly occurring events shows that the evidence for periodicity is weak, but that as found by the autocorrelation analysis, this weak evidence points towards 64 days as a possible period.

A side effect of the simulations is an estimate of about 300 dimming events in total over the period between 11 June 2004 and 21 February 2015 (3851 days). On average, an event therefore occurs about once every 13 days, and there are 30 dimming events each year (and roughly 15 per observing season). If events repeat three times, then a *new* dimming event would appear every 39 days on average, but we would also expect to see two other unrelated events during this time.

The total is five times larger than the 60 events detected by the iterative search. Most of these events were therefore either missed by the WASP and KELT-North observations or not counted because they were misidentified as single events. From figure 1, a rough estimate is that three in five events would have been missed owing to incomplete temporal coverage, meaning that about one in five simulated events were sufficiently close to other events that they were not separately identified. Not all events are deep, but as could be surmised from figure 1, near continuous observation of RZ Psc would yield a rich light curve. If continuous coverage allowed closely occurring events to be separated, then the number identified in a given time period would approximately double.

#### 4.1.3. Summary of period search

We found a weak periodic signal near 60–70 days, but neither of the methods described above show compelling evidence that the dimming events seen towards RZ Psc are periodic and not random. While we presented the results for single seasons’ data, we saw no evidence for periodicity on longer periods. Aside from the 12 year variation, no periodicity has been seen in the past [40]. These searches used periodograms, which are sensitive to variations with fixed phase and poorly motivated, so we explored autocorrelation and a similar method. We found a possible signal that can be attributed to both a different method and the significantly better temporal coverage of the WASP and KELT-North data.

A lack of strong evidence for periodicity is perhaps surprising, because material that occults the star once and is on an unperturbed orbit must pass in front of it again. Not all material need return at the same time however, and the prediction of the shearing estimate made at the outset, that the visible lifetime of clumps when they are optically thin is similar to the orbital period, appears to be borne out. Of course, shearing is not the only possible explanation, as pressure effects in a hydrodynamic turbulence scenario might also disperse a clump (as the sound crossing time for a star-sized clump near 1 AU is of order or shorter than an orbit). The latter scenario relies on a significant gas reservoir, so the primary test to distinguish between different clump scenarios lies with the evolutionary status of the disc, which we explore in §5.3.

### 4.2. Gradient analysis

Given the possibility of a 60–70 day periodicity, the location of the occulting bodies could be relatively close to the star, with semi-major axes of about 0.3 AU. This distance is comparable to the 0.4 AU estimated for optically thin dust at 500 K [40]. To further investigate the location, we turn to a different aspect of the light curves that provides information on the velocity of the occulting bodies: the gradients. To convert gradients measured in the light curves to velocity and orbital distance, we first outline a simple model, and then use this model to interpret the data.

#### 4.2.1. ‘Curtain’ model

This section considers a simple one-dimensional model (along *x*) of a cloud that dims a star. The main assumption is that the cloud is larger than the star, so for a cloud that passes in front of the star from left to right, the vertical (*y*) size of the cloud can be ignored. The large cloud extent is not only suggested primarily by the large depths of the dimming events, but also because no flat-bottomed (i.e. planet transit-like) events are seen. It seems likely that not all clumps are this large, and that a variety of sizes (and impact parameters) exist, but for our purposes this simplification is sufficient. Thus, the cloud is modelled as a semi-opaque screen or ‘curtain’ that dims the star, as in previous analyses of related phenomena (e.g. KH-15D, J1407 [54,69,70]).

The star is dimmed by the passage of a cloud located at *x*_cl_ from the star centre. The one-dimensional geometrical optical depth structure of the cloud is given by some function centred at *x*_cl_ (e.g. a top hat or Gaussian) so is *τ*(*x*−*x*_cl_). The star has a surface brightness $I(\sqrt{x^{2}+y^{2}})$, which could allow for limb-darkening. The observed flux from the star is then

4.2 $$F(x_{\mathrm{cl}})=\int_{-R_{⋆}}^{R_{⋆}}(1-\tau (x-x_{\mathrm{cl}}))\;dx\int_{-\sqrt{R_{⋆}^{2}-x^{2}}}^{\sqrt{R_{⋆}^{2}-x^{2}}}I\left(\sqrt{x^{2}+y^{2}}\right)dy,$$

which first integrates vertically over the star at some *x* (i.e. independently of *τ*), and then along *x*, which includes the effect of the cloud. The light curve is therefore the convolution of the one-dimensional stellar brightness profile with the clump’s optical ‘thin-ness’ profile (i.e. 1−*τ*). The flux profile (light curve) is a function of time, but the star and clump profiles are functions of *x*, and the conversion that links these is the cloud velocity.

The simplest case is a star of uniform surface brightness that is occulted by an optically thick screen that covers the star from *x*=−1 to *u* (i.e. the units of length are now *R*_⋆_). Then, *I*=1 and *τ*(*x*−*x*_cl_) is a step function at *u* and the fraction of the total stellar flux (*F*_⋆_=*π*) seen is [69]

4.3 $$f=\frac{F}{F_{⋆}}=\frac{\left(\cos^{-1}[u]-u\sqrt{1-u^{2}}\right)}{\pi },$$

where *f* has the same units as our normalized light curve. If the curtain is not completely optically thick, then the fraction is instead

4.4 $$f=\frac{F}{F_{⋆}}+\left(1-\frac{F}{F_{⋆}}\right)(1-\tau ).$$

The gradient of the normalized light curve as the curtain is pulled across is *df*/*du*, and therefore the sky-projected (i.e. minimum) velocity of a clump is

4.5 $$\frac{du}{dt}=-\frac{\pi }{2\tau \sqrt{1-u^{2}}}\;\frac{df}{dt}.$$

A cloud that is not completely optically thick has a shallower flux gradient because it reaches a shallower depth for the same velocity, and the factor 1/*τ* accounts for this effect. Stated another way, for this curtain model the optical depth and cloud velocity are degenerate in producing some flux gradient. However, this degeneracy can be partially broken, because some information on *τ* exists; *τ* must be greater than the depth of the dimming event (i.e. 1−*f*_min_, the minimum normalized flux). Objects somewhat smaller than the star are also accounted for; an optically thick clump that covers half the star produces approximately the same light curve as a *τ*=0.5 clump that covers the whole star.

These expressions can be further simplified by assuming that the maximum gradient occurs as the cloud ‘edge’ passes the centre of the stellar disc (i.e. *u*=0). By adopting a radius for the star, this velocity can be converted to physical units, and assuming a stellar mass and that the clump is on a circular orbit converted to a semi-major axis. If *df*/*dt* is in units of fractional stellar flux per day (i.e. the light curve is normalized and has time units of days) and *du*/*dt* in stellar radii per day (i.e. units of *u* are *R*_⋆_), then the numbers for these quantities can be used in the following equations

4.6 $$v\approx 8050\frac{R_{⋆}}{R_{⊙}}\;\frac{du}{dt}{m\;s}^{-1}$$

and

4.7 $$a_{\mathrm{circ}}=14\frac{M_{⋆}}{M_{⊙}}{\left(\frac{R_{⊙}}{R_{⋆}}\;\frac{dt}{du}\right)}^{2}\mathrm{AU}=8.7\frac{M_{⋆}}{M_{⊙}}{\left(\tau \frac{R_{⊙}}{R_{⋆}}\;\frac{dt}{df}\right)}^{2}\mathrm{AU}.$$

While the assumption of *u*=0 yields a simple conversion between the light curve gradient and the velocity and semi-major axis, it is of course possible to measure gradients that are not at *u*=0. For example, the gradient when a dimming event reaches minimum is zero, which implies zero velocity and an infinite semi-major axis (e.g. the first minimum in 2006 in figure 2). In addition, orbits may not be circular and thus the actual velocity is greater than the sky-projected velocity during a dimming event. Thus, the gradients and velocities must be taken as lower limits, and the semi-major axes as upper limits.

#### 4.2.2. Curtain model application

**Figure 7.**
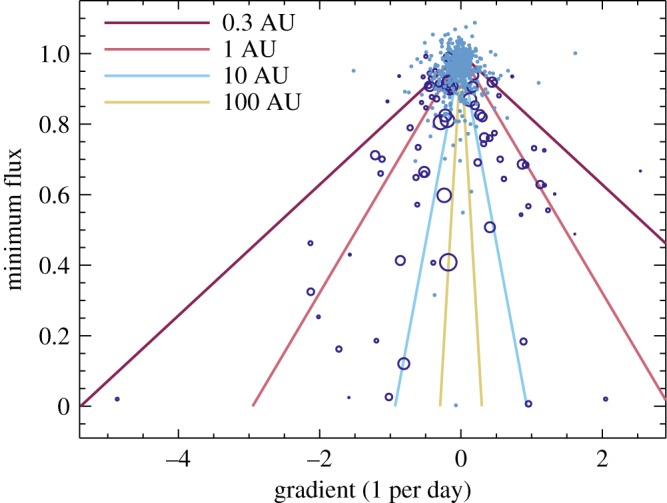
Gradient and minimum flux measured from individual nights’ observations. Open circles have gradients significantly different from zero, and a symbol size proportional to the inverse of the gradient uncertainty. Dots are consistent with zero slope. The lines show the gradients implied by the velocities for circular orbits at 0.3, 1, 10 and 100 AU.

Using the simple formalism described above, we can use gradients derived directly from the light curves to estimate the radial location of the occulting clumps. The gradients are estimated by least-squares fitting straight lines to each night’s observations, for which only nights with six or more measurements are used. This procedure is possible, because in nearly all cases, an individual night’s observations only cover ingress or egress, not both. These gradients are plotted against the minimum nightly flux *f*_min_ in figure 7. We plot gradients whose uncertainty is less than 4× their value as open circles, with symbol sizes proportional to the inverse of this uncertainty. All other gradients are plotted as small dots, as a check that the gradients for unocculted fluxes are near zero. The horizontal scatter of these gradients near *f*_min_=1 provides a further estimate of the uncertainty of individual gradients. The solid lines show the gradients expected for circular orbits at a range of semi-major axes.

An unusual feature in figure 7 is that the gradients may be biased towards negative values at low *f*_min_. For the 15 negative, and four positive gradients below *f*_min_=0.5, and approximately equal numbers of positive and negative gradients above, Fisher’s exact test yields a *p*-value of 0.02. Thus, there is evidence that the distributions of gradients above and below minimum fluxes of 0.5 are different, with negative gradients more commonly seen for deep dimming events. In the range 0.5<*f*_min_<0.8, there are 15 and 25 negative and positive gradients, a reversal of the trend, but this difference only has a *p*-value of 0.1. Thus, there are about equal numbers of gradients measured with minimum fluxes below 0.8, but their distributions are different.

The bias to negative gradients below *f*_min_=0.5 suggests that ingress tends to be slower than egress; the egress is too quick to be caught. However, the equal numbers between 0.5<*f*_min_<0.8 suggest that the rapid egress does not return the light curve to quiescence, just to a level above *f*_min_=0.5. Thus, the statistics suggest that a typical deep dimming event has an ingress at a rate of −1 to −2 day^−1^, after which the flux rapidly rises to *f*_min_≈0.5, and then the remaining egress is at a rate similar to ingress.

Qualitatively, this inference is consistent with a scenario of a disrupted asteroid, whose structure is dictated by shear and radiation pressure. The fragment size distribution is such that at least half of the optical depth is contributed by grains large enough that their orbits relative to the original body are dominated by shear; forward shearing is more rapid than backward shearing, so the clump has a sharper rear edge than front edge, accounting for the different number of positive and negative gradients measured for *f*_min_<0.5. The fragments also comprise small grains whose dynamics are dominated by radiation pressure, which form a ‘tail’ much like a comet’s and account for the egress where *f*_min_>0.5. We leave the development of a quantitative study of this scenario for the future, noting that tests of such a model would require photometry at multiple wavelengths.

A look at specific dimming events shows that such a simple scenario will face challenges, as shown by the first set of events in 2006 (figure 2); following the *f*≈0.5 dip, there are two more nights of data that have higher average fluxes, but both nights actually have negative gradients. This evolution does not invalidate the above analysis, but shows that the temporal evolution is complex, and at any given time multiple clumps, which may or may not be related, could be occulting the star.

**Figure 8.**
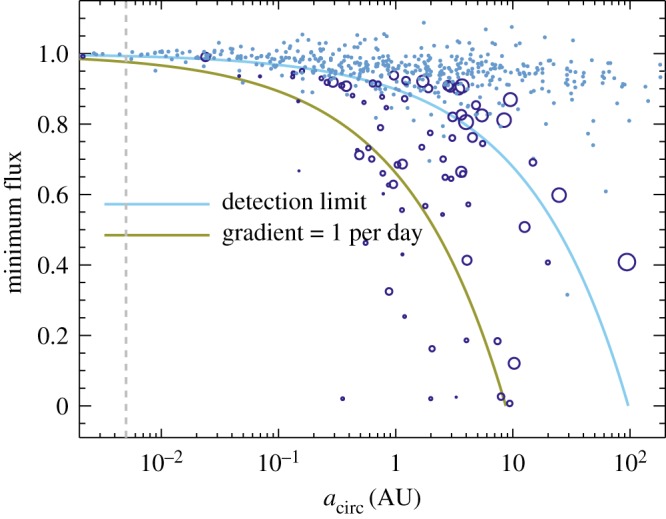
Semi-major axes estimated from the light curve gradients in figure 7. Open circles have gradients significantly different from zero, and a symbol size proportional to the inverse of the gradient uncertainty. Dots are consistent with zero slope. The stellar radius (grey dashed line) has been estimated as solar. Because gradients may have lower minimum fluxes, they can move down along lines parallel to the solid lines. Points near or above the blue line are consistent with zero light curve gradient.

In figure 8, we have converted the gradients into semi-major axes using equation (4.7). The dashed line shows the stellar radius, estimated to be approximately solar based on an age of 25 Myr [71] and an effective temperature of 5350 K [45], using Siess *et al.’s* [72] isochrones. Figure 8 also includes solid lines of constant gradient, computed using equation (4.7) and *f*_min_=1−*τ*.

The blue line shows the semi-major axis implied by the scatter in gradients near *f*_min_=1 in figure 7. Above this line, the gradients can be considered consistent with zero, and the inferred semi-major axes largely meaningless. A related point is that because the points near *f*_min_=1 have very small *τ*, the derived velocities and semi-major axes can be rather extreme and should be disregarded. An additional issue is highlighted by the two large circles near *f*_min_=0.5 and between *a*_*circ*_=30 and 100 AU, which are the first two nights of 2006 observations shown in figure 2. The first is at the time of minimum flux, and the other also appears to be at a turning point, so the assumption behind equation (4.7) that *u*=0 (i.e. the cloud edge is passing the stellar disc centre) is clearly incorrect. These points should therefore be associated not with clumps at 30–100 AU, but with clumps that lie somewhere interior.

The green line shows the semi-major axis implied by a gradient of 1 per day. As the lowest flux measured on a given night is not necessarily the minimum flux for that dimming event, the inferred semi-major axis could be greater; in this case, points move downward parallel to this and similar lines. Figure 8 suggests that the clumps orbit with projected velocities that are consistent with circular orbits at or beyond about 1 AU. The conclusion from the IR excess, and possibly the periodicity analysis, is that they may lie closer, near 0.3–0.7 AU. Reconciling these differences requires that the clumps are on eccentric obits. For a semi-major axis of 0.3 AU, the minimum clump eccentricity (i.e. all clumps transit at apocentre) is about 0.6. Such an orientation is of course highly unlikely, so either the semi-major axis is larger, or the true eccentricities need to be higher. Perhaps coincidentally, an eccentricity of 0.6 yields a pericentre velocity of 120 km s^−1^, similar to the velocity shifts in sodium absorption lines seen towards RZ Psc, which are discussed further in §5.2 (noting, however, that the pericentre velocity is tangential, and the absorption lines show projected radial velocity [73]).

### 4.3. Summary of light curve analysis

The aim of this section was to estimate the location of the bodies that cause the dimming events towards RZ Psc. Using 10 years of WASP and KELT-North data, we investigated the periodicity of possible repeat events and the light curve gradients. While we found evidence for a 60–70 day period, this signal is weak and not statistically significant. This period would place the clumps near 0.3 AU, similar to the distance inferred for the dust seen as an IR excess (though this location is also uncertain). The gradient analysis places loose constraints on the clump orbits (less than 10 AU), so is consistent with a scenario where the clumps have semi-major axes near 0.3 AU. Thus, based on the light curve analysis, there is no reason to disfavour the model proposed by de Wit *et al.* [40], that the dimming events are associated with clumps being created by planetesimal collisions within an asteroid belt analogue.

The primary uncertainty lies with the disc evolutionary state. If a significant gas reservoir remains, a turbulent inner rim scenario similar to that proposed for UXors might produce a similar light curve. The lack of accretion and a likely age beyond which gas-rich discs are typically seen, may argue against this scenario, though the disc may be in transition to the debris phase and retain some primordial gas. We revisit the disc status from the perspective of the flux distribution in §5.3.

An additional result from the gradient analysis concerns the structure of individual clumps. The non-uniform gradient distribution is qualitatively consistent with post-collisional asteroidal fragments being dispersed by a combination of shearing and radiation pressure. Assuming small dust well-coupled to gas in a hydrodynamic turbulence scenario, a uniform gradient distribution seems more likely, so we interpret the gradients as providing circumstantial evidence for the planetesimal fragment scenario.

## 5. Disc structure and evolutionary state

Based on 10 years of relatively high-cadence photometry, RZ Psc is regularly occulted by what are almost certainly star-sized clumps of dust. These clumps can be optically thick, and previous measurements of colour variations show that at least some of the dust must be small [38], which may be supported by the light curve gradient statistics. The previous interpretation of this system was that the clumps are the fragments arising from planetesimal collisions within an asteroid belt analogue that is also detected in the mid-IR. While the results from the previous section are consistent with this scenario, the evidence is at best circumstantial as its does not rule out the alternative of a UXor-like hydrodynamic inner rim scenario.

We now address several open questions, each taking a slightly wider view. Primary among these is whether the dimming events and the IR excess are caused by the same dust, as suggested by de Wit *et al.* [40]. Two further aspects are then the implications for the origin of the clumps and the evolutionary status of the disc in which they reside. We finish by considering the proposed scenarios for dippers and UXors, and why RZ Psc appears to be a rare object that lies between these classes.

### 5.1. Does the occulting dust account for the IR excess?

One of the reasons that RZ Psc is worthy of detailed study is that circumstellar dust is inferred from both the dimming events and the IR excess. Different properties of the dust grains, and the larger structure in which they reside, are revealed by each method; the dimming events yield information on dust ‘clumpiness’ on a star-sized scale, whereas the IR excess provides evidence for a disc that captures approximately 7% of the starlight, and thus a measure of the total surface area of dust. The proposed interpretation is that the clumps orbit within an asteroid belt, and the dimming events therefore provide some information on the size distribution and collisional evolution within the belt [40]. This expectation relies on co-location of the clumps and the belt, for which circumstantial evidence is provided by the light curve gradients and perhaps the periodicity analysis (see also [39]).

The fraction of starlight intercepted by the dust is a variable common to the dimming events and the IR excess. For the former, we use the average extinction $\bar{E}$, which is simply taken from the normalized light curve, as 1 minus the average flux, yielding 0.05 (the light curve median is 0.995). This estimate assumes that all dimming events are independent, and the value would be smaller if not, because the dust in some clumps may be being counted two or more times. The lack of strong evidence for periodicity suggests that multiple counting is not a serious issue however. Another issue is that the star could be reddened, and therefore that the normalized light curve has already had some constant level of extinction removed. Based on photospheric colours, this unseen extinction is probably small, in the range of zero to a few per cent [74].

If we assume that this average extinction applies over a uniform sphere around the star, and that the dust has a low albedo (i.e. the dimming events are dominated by dust absorption, not scattering of light out of our line of sight), then the IR fractional luminosity is equal to the average extinction. That is, both are equal to the fraction of starlight intercepted by the dust.

To explore possible geometries we use a simple relation between fractional luminosity *L*_disc_/*L*_⋆_, (uniform) geometrical optical depth *τ*, and the disc opening angle *θ* [12],

5.1 $$\frac{L_{\mathrm{disc}}}{L_{⋆}}=\tau \sin\left(\frac{\theta }{2}\right),$$

which says that the fractional luminosity is the optical depth of the dust multiplied by the fraction of the sky covered as seen from the star. The dust belt must therefore have an opening angle of at least 8^°^ to capture 7% of the starlight. However, for this minimal estimate, the dust is optically thick, yet RZ Psc is not seen to be reddened. If we instead require $\tau ∼\bar{E}$, then as stated in the previous paragraph, the dust distribution must instead be near isotropic. Given the ubiquity of disc-like structures around young stars, such a spherical distribution seems physically unlikely. In addition, the increased polarization during deep dimming events argues against a spherical distribution.

Thus, the picture of RZ Psc as a star seen *through* a disc, where the clumps account for all of the dust and sample some representative part of an asteroid belt (e.g. the midplane), is untenable because that belt would cause much more reddening than is observed. These characteristics distinguish RZ Psc from heavily reddened objects, where the dimming events could be sampling a more representative section of the disc [75]. This issue can be avoided by invoking a spherical distribution of material, but the problem then shifts to whether such a distribution is physically plausible.

A more likely alternative, which we favour, is that most of the dust does not lie on orbits that pass in front of the star, and the occultations are caused by a small fraction of objects that have higher vertical locations (or greater orbital inclinations) than average. In this case, the component that causes most of the IR excess may or may not be clumpy, and could be radially optically thick (i.e. the opening angle could be as small as 8^°^). This picture unfortunately loses any strong connection between the occulting clumps and the IR excess, essentially adding a free parameter that is the fraction of material that is ‘kicked’ or resides above the disc, but seems to be the simplest and most probable scenario. Dullemond *et al.* [33] used essentially the same argument for UXors, so our picture is therefore inevitably similar to dust occultation models proposed for UXors [22,33,76]. As the disc is probably radially optically thick with a scale height similar to gas-rich protoplanetary discs, it could be that the scenario for RZ Psc is in fact the same as proposed for UXors. In this case, the IR excess would originate from the inner edge of a more extended disc, which is not detected at longer wavelengths for reasons discussed below.

We therefore conclude that while there is almost certainly some connection between the dimming events and the IR excess, it is at best indirect; we are not viewing RZ Psc through a representative part of an asteroid belt analogue. As with other UXors, a clear prediction is that the disc is not seen edge-on, but at an intermediate inclination.

### 5.2. Origin of the occulting structures

One of the distinguishing characteristics for RZ Psc is the relatively short duration of the dimming events *t*_dim_, which are a few days compared with a few weeks for other UXors [21,77]. If we assume near-circular orbits at speed *v*_kep_, *t*_dim_=2(*R*_cl_+*R*_⋆_)/*v*_kep_, where the clump has radius *R*_cl_. Solving for the clump radius yields the relation [32,78]:

5.2 $$R_{\mathrm{cl}}\approx 1.85t_{\dim}{\left(\frac{M_{⋆}}{M_{⊙}}\frac{1\mathrm{AU}}{a}\right)}^{1/2}-R_{⋆},$$

where here *R*_cl_ and *R*_⋆_ are in units of *R*_⊙_ and *t*_dip_ is in days. Equation (5.2) says that dimming events of a given duration can, in general, be caused by larger clumps that orbit close to the star or smaller clumps that orbit farther out.

**Figure 9.**
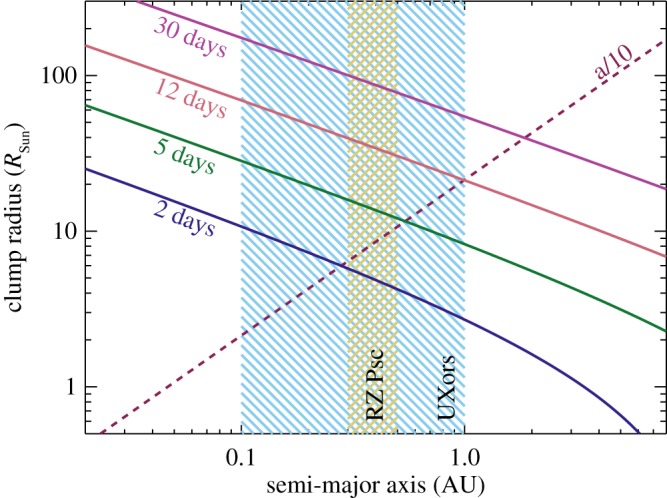
Clump properties assuming circular orbits for a range of dimming event durations (as labelled). The brown region marks the approximate location of the RZ Psc asteroid belt (or the inner edge of a more extended disc). The blue-shaded region shows the range of Herbig Ae inner disc edge radii. The dashed line shows where a clump has an azimuthal extent similar to the scale height of a typical gas-rich disc.

Figure 9 shows the radius that clumps must have to cause dimming events of different durations as a function of semi-major axis. For clumps much larger than the star the stellar radius is unimportant, and the stellar mass dependence is relatively weak, so this plot can be applied to RZ Psc and UXors. The radius is of course the sky-projected size of a clump along the orbit, so whether this scale also applies vertically and radially depends on the specific scenario. This plot assumes circular orbits, and that the star has solar radius and mass. The approximate locations of the RZ Psc belt (or the inner edge of a more extended disc) and the range of inner edge radii for Herbig Ae stars [35] are shown by the hatched regions.

The dashed line shows where clumps extend a tenth of the semi-major axis—approximately, the scale height for a gas-rich disc. If the variability of both UXors and RZ Psc originates from dust structures arising from hydrodynamic turbulence at the disc inner edge, and these structures are related to the disc scale height, then a wide range of dimming event times is expected. These times should correlate strongly with the inner edge location, and because this location is set by sublimation [35] should correlate with the luminosity of the star. We did not find evidence for such a correlation in time-variability studies of UXors [79], suggesting that the clump radii do not vary strongly with the inner edge location. In any case, we have already noted that RZ Psc has shorter dimming events than ‘typical’ UXors, so while RZ Psc has dust at a radius that falls within the range of UXors, the occulting clumps are inferred to be several times smaller.

A further difference between RZ Psc and UXors is the origin of the dust location. For Herbig Ae/Be stars (and by extension, UXors), the dust inner radius is set by sublimation. However, the dust around RZ Psc is roughly 500 K, so much cooler than the approximately 1500 K sublimation temperature. That is, if the origin of RZ Psc’s variability is interpreted as similar to other UXors and originates in a gas-rich disc, then it must host a transition disc rather than host a ‘full’ primordial disc. Therefore, while these comparisons show RZ Psc to be unusual compared with typical UXors, they do not argue strongly for or against a specific scenario.

A final aspect to discuss regarding the origin of the clumps, and their relation to the IR excess, is the transient absorption features. These are seen towards UXors, but also seen towards some main-sequence A stars [80–82], so are not exclusive to stars that host gas-rich discs. For A-type stars, these features are generally interpreted as Sun-grazing ‘exocomets’, and the same may apply to UXors and RZ Psc. A potential issue with this interpretation is that the absorption lines towards RZ Psc are so far blue-shifted, and may instead originate in an outflow [73]. However, blue-shifting could also occur if evaporation mostly occurs near periastron passage, which might be expected if the bodies originate in an asteroid belt and are thus more refractory than the exocomets seen towards other stars (i.e. an asteroid in the Solar System would need to pass very close to the Sun to have a tail). Such a scenario is also consistent with the conclusion of §4 that the occulting clumps could be on eccentric orbits with high velocities at periastron. Models of such low-periastron asteroids or comets invariably require a perturbing planet [83,84]. Assuming the same 4 : 1 mean-motion resonance picture of Beust & Morbidelli [84] implies a planet about 2.5 times more distant than the source. That is, if the asteroid belt is at 0.3 AU, the planet is near 0.75 AU. It may be possible that this planet is inclined and causes the asteroid belt to precess, thus causing the 12.4 year modulation of the stellar flux seen by de Wit *et al*. [40].

Overall, the origin of the clumps remains unclear, and raises many further questions. A distinguishing feature of RZ Psc’s light curve is that most of the time the star is near the quiescent level; if the dimming events are related to variable structure at the inner rim of a gas-rich disc, then why are the events so rare? Is the geometry so finely tuned that only the most extreme fluctuations are visible? In the asteroid belt scenario, the details are equally unclear; if the clumps are recently disrupted planetesimals, how do they appear above the bulk of the disc when collisions are most likely to occur at the midplane? Are there two populations of objects, one that forms the main disc, and another that causes the dimming events? Could such a scenario be reconciled with the transient absorption features? Answers to these questions will require further study, and will almost certainly require new observations.

### 5.3. Disc evolutionary state

Many young stars with gas-rich discs show broadly similar variability (UXors, AA Tau analogues, dippers). With sporadic and deep photometric minima, and transient absorption lines, RZ Psc is most similar to UXors and has often been associated with this class, albeit as an unusual member [39]. Initial distinctions were made based on the late spectral type (K0V), the relatively short dimming events, and a lack of near-IR excess and accretion signatures. The more recent findings that the stellar age is probably several tens of Myr, and that the IR excess is suggestive of an asteroid belt analogue, further distinguish RZ Psc as a potentially remarkable object where the early gas-poor stages of main-sequence debris disc evolution can be studied. There remain similarities between RZ Psc and UXors. Primarily, we concluded that the clumps that cause the dimming events are most likely seen when they are well above the densest regions of a disc, consistent with the turbulent inner rim scenario proposed by Dullemond *et al.* [33].

**Figure 10.**
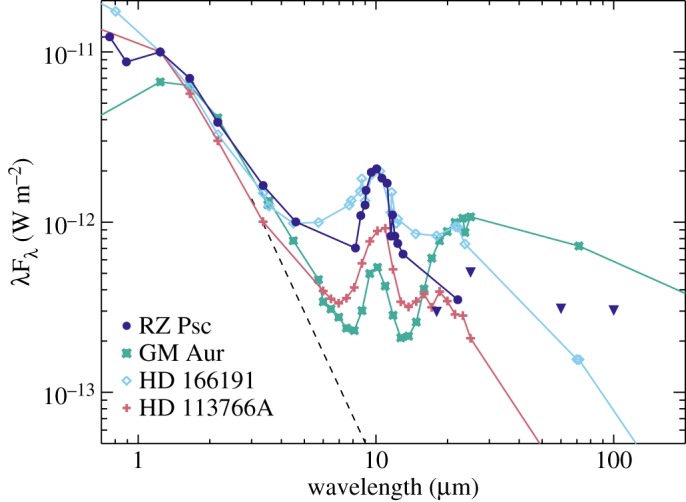
The spectral energy distribution of RZ Psc in comparison with other young disc-hosting stars. All SEDs are normalized to a common flux density at *H* band. The triangles are AKARI and IRAS upper limits for RZ Psc. Measurements were made at different times, so the apparent discrepancy between the WISE detection at 22 μm and the AKARI upper limit at 18μm is an indicator of IR variability (see §3.2).

To consider RZ Psc within the context of other young disc-hosting systems, figure 10 shows the spectral energy distribution (SED) of RZ Psc, and several other systems that could be considered to be at a similar evolutionary stage (a similar plot appeared in [12]). GM Aur hosts a transition disc [85], the status of HD 166191’s disc is ambiguous and may lie somewhere between the transition and debris phase [12,86], and HD 113766A is generally considered to host a bright warm debris disc (based on a 10–16 Myr age and a lack of gas [87], but based on similarities with HD 166191 was also noted as potentially ambiguous [12]).

In contrast to UXors, there is no obvious reason for dust around RZ Psc to lie near 0.3 AU, as the dust sublimation distance is much closer. For RZ Psc to host a gas-rich disc, it would therefore probably need to be a transition object that has as-yet undetected far-IR emission from the outer disc. As illustrated by figure 10, the limits set by IRAS are not particularly stringent, but in comparison with GM Aur the SED beyond 20 μm is a factor of two lower, despite the mid-IR SED being a factor of two brighter. Transition discs have a wide variety of spectra however [58,88], so the main conclusion from this comparison is that if an outer disc exists, it is not very bright. This far-IR deficit is a signature of self-shadowing, which is of course a key characteristic of UXors. If such a picture were true for RZ Psc, a prediction is that the outer disc may still be detectable at millimetre wavelengths (roughly mJy levels), whereas an extrapolation based on the asteroid belt scenario would not (roughly μJy). Specifically, self-shadowing can be caused by settling of dust towards the disc midplane, which can occur with little loss of vertical optical depth [89]. Thus, the far-IR flux can be much lower than for a typical disc, whereas the mm-wave flux is not.

In figure 10, RZ Psc looks more similar to HD 166191 and HD 113766A, thus falling in the category of systems whose interpretation in terms of disc status is ambiguous (the high fractional luminosity would also mark RZ Psc as unusually extreme for a debris disc). With the aid of mid-IR interferometry, the disc around HD 113766A has been shown to comprise two components, one at 0.6 and another at 9 AU [90]. This is by no means evidence that RZ Psc has a similar structure, but merely reinforces the fact that the SED does not rule out such possibilities and that the dust need not be confined to a single belt near 0.3 AU, and need not be interpreted as an asteroid belt. Similarly, the disc around HD 166191 was modelled as an optically thick transition disc extending from 1 to 25 AU [12]. Thus, as was concluded above by considering self-shadowing, it could be that RZ Psc hosts a disc that extends from 0.3 to a few tens of AU.

While the lack of a large near-IR excess for RZ Psc suggests that the disc is at least in the transition to a debris disc (i.e. has an inner hole), it does not preclude the possibility that gas resides in that hole and may still be accreting onto the star. No emission lines that would provide evidence of accretion have been seen [45,73], but as a further test we reconsidered the spectral energy distribution. Specifically, we included photometry from the galaxy evolution explorer (GALEX [91]) to quantify the level of any ultraviolet (UV) excess, a complementary accretion indicator [92]. This exercise is hindered somewhat by the possibility that optical photometry was obtained when RZ Psc was not near the quiescent level. To circumvent this issue, we used just the 2 μm all-sky survey (2MASS [93]) and GALEX photometry, fitting a PHOENIX atmosphere model [94], finding a best-fit effective temperature of 5485 K (assuming no reddening, or 5600 K if some reddening is allowed to improve the fit slightly). These temperatures are consistent with that derived from a high-resolution spectrum; 5350±150 K [45]. Alternatively, fixing the temperature to the spectroscopic value yields a mild UV excess, less than a factor of two, that may be chromospheric. We therefore conclude that there is no evidence for accretion seen as a UV excess.

Finally, the mid-IR variability discussed in §3.2 provides a measure of the changing emitting area of the disc around RZ Psc, and thus potentially information about the disc structure and status. Figure 4 shows that for the epochs where contemporaneous 3–22 μm photometry exists there is no evidence of ‘see-saw’ variability over six months, but that strong conclusions are limited by a lack of data. Over 5 years, the 3–5 μm disc flux varied by about a factor of two, with no major changes in the behaviour of the optical light curve. However, given that the bulk of the disc emission probably originates from material that is not occulting the star, direct links between the optical and IR behaviour are not necessarily expected. Indeed, towards young stars hosting gas-rich discs, correlated and uncorrelated optical/IR variability is seen [17]. Similarly, the IR flux of bright warm debris discs has been seen to vary strongly, whereas the optical brightness remains constant [95]. The main benefit of more intensive IR monitoring would be to search for mid-IR ‘see-saw’ variability, because it would provide evidence that the disc around RZ Psc has a significant radial extent.

In summary, the status of the disc surrounding RZ Psc is unclear. Despite a reasonable near/mid-IR characterization, at longer wavelengths the SED is not detected. Comparison with the IR spectra of other discs suggests that the disc is at a minimum well evolved towards the debris phase, and may have reached it already. This possibility, and the rarity of objects such as RZ Psc, opens the possibility that it is being observed at a special time with a specific geometry, so may yield better insights than most systems.

### 5.4. The rarity of Sun-like dippers/UXors

**Figure 11.**
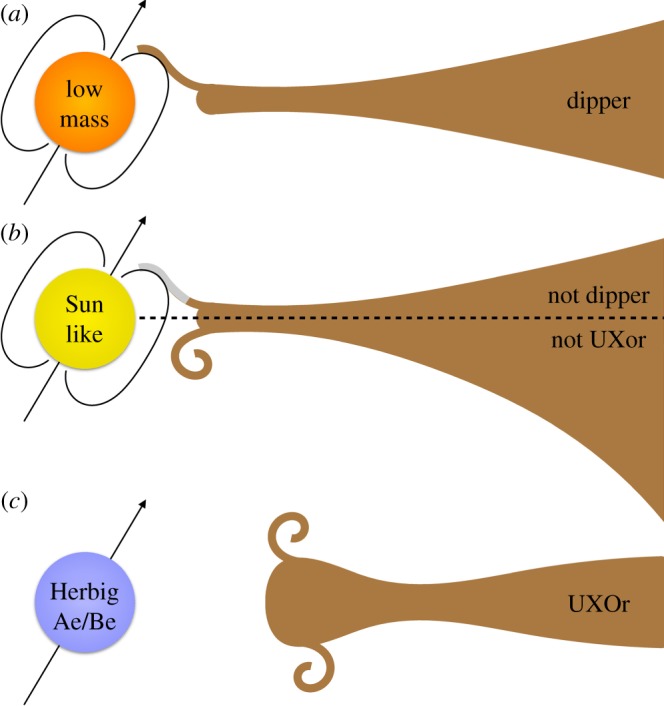
Cartoon shows possible origins of dippers and UXors, and why Sun-like stars may only rarely show analogous behaviour. In each case the star, magnetic dipole and rotation axis are shown at the left (the stellar magnetic field is not necessarily always tilted with respect to the disc). Possible disc structures viewed edge-on to the right. (*a*) Low-mass stars (dippers) are occulted by co-rotating material that is accreting onto the star, and the dust sublimation radius is interior to co-rotation [34]. (*b*) Sun-like stars are rarely seen as dippers or UXors because (i) dust sublimates outside co-rotation (represented by the grey accretion column in the upper half [34]) or (ii) material lifted by turbulence is shadowed by the outer disc (spiral in the lower half). (*c*) Herbig Ae/Be stars (UXors) are occulted by turbulence that appears above self-shadowed discs [33].

We finish by briefly discussing RZ Psc in the context of the general classes of dippers and UXors, considering why neither of these classes includes many young Sun-like stars such as RZ Psc. To aid this discussion, figure 11 shows simplified cartoons of the proposed scenarios for dippers and UXors, and possible reasons for a lack of significant dust-related dimming events towards Sun-like stars (see [34] for similar figures for dippers).

From the perspective of dippers, which so far have spectral types later than K5, Bodman *et al.* [34] explain their tendency to be low-mass stars as a consequence of the relation between the magnetospheric truncation, co-rotation and sublimation radii. The periodicity of dippers suggests that the occulting material is near the co-rotation radius, and is therefore near the base of any accretion columns. For low-mass stars, the dust temperature at this distance is cool enough that the columns contain significant dust mass and hence the dipping phenomenon is seen (figure 11*a*). For earlier type stars, the sublimation radius is outside the co-rotation radius, so any dust in the accretion columns has sublimated, and no dipping is seen (grey accretion column in figure 11*b*).

An as-yet unexplored corollary of this scenario is that the outer discs in dipper systems must not be more flared than the height of the accretion columns (as seen from the star). This could be because discs around low-mass stars are simply less flared in general [96], because discs in dipper systems tend to be more evolved than average [32] or because there is less relation between the outer disc geometry, the inner disc and accretion columns than would be naively expected [97]. Of course, if discs around Sun-like stars are sufficiently flared that the inner regions are not visible, then whether the accretion streams are transparent or not is moot.

From the perspective of UXors, a possible explanation for their tendency to be late B- and A-type stars is that the specifics of self-shadowing are different for Sun-like stars [33]. There is little reason to believe that self-shadowing does not happen for young Sun-like stars [89,98], so it seems either that self-shadowing is simply rarer, or that the nature is different in a way that affects whether the inner disc can occult the star (i.e. shadowing is by a larger portion of the inner disc regions rather than by a puffed-up inner rim [89,99]).

Thus, it seems that the rarity of Sun-like stars among dipper and UXor populations may be understood as a result of the scenarios for both. Accretion columns are optically thin, because the dust has sublimated, and any turbulence that rises above the inner disc may not be seen as it is hidden behind a flaring outer disc.

In this context, the rarity of RZ Psc-like objects can be explained in two ways. The first simply sidesteps the above discussion by interpreting the disc as a gas-poor asteroid belt analogue. Examples of such bright discs at a few AU are rare [10], and only a subset of these will be oriented such that dimming events are seen above the disc midplane (recalling that the occulting bodies are at tens to hundreds of stellar radii). The second explanation relies on RZ Psc’s age and SED, which suggest that it hosts a gas-rich transition disc that is well settled (i.e. not significantly flared), and hence turbulence above the disc inner edge is visible. The non-detection of RZ Psc in the far-IR (figure 10) might also be the result of such settling, and suggests that the brightness at millimetre wavelengths might be brighter than expected given the mid/far-IR brightness [89,99]. The rarity is again explained by an unlikely geometry, and perhaps that the period during which the inner disc can be seen above the outer disc as it settles is relatively short.

## 6. Summary and conclusions

Long considered a member of the UXor class of variables, RZ Psc is almost completely occulted by dust for several days, multiple times during each observing season. The ‘typical’ UXor (which is generally a Herbig Ae object) shows day to month long dimming events that are thought to be caused by hydrodynamic turbulence above the disc inner rim, and where the outer disc is self-shadowed [33]. Various anomalous characteristics distinguish RZ Psc from other UXors; the possible age of a few tens of Myr, the K0V spectral type, the few-day long dimming events and the location of the IR-excess emitting dust well beyond the sublimation radius. These characteristics have been used by de Wit *et al.* [40] to suggest that RZ Psc hosts a gas-poor asteroid belt analogue at 0.4–0.7 AU and that the dust clumps that occult the star are the dispersed fragments produced in destructive planetesimal collisions.

To take a critical look at this intriguing scenario, we have presented and analysed 10 years of WASP and KELT-North photometric monitoring of RZ Psc. We found circumstantial evidence that some dimming events repeat and have a semi-major axis consistent with that inferred from the IR excess, but the signal is not significant (1–2*σ*). The light curve gradients are consistent with this picture, but the constraints are poor. The statistics of the light curve gradients suggest that a typical dimming event has an egress rate that is initially faster, and then slower, than ingress. While this evolution seems qualitatively consistent with the structure expected from a planetesimal collision, quantitative models are needed.

By considering the joint constraints allowed by the light curve and the IR excess, we find that the objects causing the dimming events are unlikely to be representative of the structure causing the IR excess. The two can only be reconciled if the IR excess originates in a spherical shell of clumpy but on average optically thin dust, a scenario disfavoured by the increased polarization during deep dimming events. Assuming a disc-like structure, the belt is almost certainly optically thick with an opening angle of a few tens of degrees, with the system viewed at an inclination that allows clumps residing above this belt to pass in front of the star. While such a geometry is possible for both UXor-like and asteroid belt scenarios, the relatively cool temperature of the dust around RZ Psc means that if it hosts a gas-rich disc it must be a transition object. Indeed, comparison of RZ Psc’s spectrum with other objects suggests that it is similar to objects whose status is ambiguous, and could be either debris or transition discs. The low far-IR disc luminosity could arise if the outer disc is shadowed (as suggested for UXors), or simply, because there is no outer disc. The spectrum is poorly sampled and would benefit from millimetre photometry, specifically to test whether there is a settled outer disc.

Overall, we conclude that the status of RZ Psc’s disc is uncertain, and therefore that so is the origin of the clumps. The lack of near-IR excess shows that the disc is beyond the primordial phase, but could be in the final throes of dispersal and the occulting structures a related phenomenon. Several specific observations would help: (i) mid-IR spectral monitoring would allow comparisons with transition disc systems that show ‘see-saw’ variability, (ii) continuous photometry (ideally with multiple colours) would yield the detailed shape of individual dimming events and in some cases the distribution of dust size across the clump.

As a young Sun-like star showing disc-related stellar variability, RZ Psc is a rarity. The reason may be twofold: (i) the sublimation radius is greater than for low-mass stars, so any accretion streams are transparent and/or (ii) in contrast to more massive stars, turbulence above the inner rim may be shadowed by the outer disc. While the asteroid belt scenario avoids the need to consider primordial disc structure, in the context of such models, RZ Psc-like variability could be explained by the evolved state of the disc, which may have settled enough that the inner rim is visible.

## Footnotes

1 Not all stars occulted by dust are UXors. Two other classes are: those occulted by circumstellar material beyond ≳10 AU, such as AA Tau and V409 Tau which show ≳year-long dimming events [23,24], and systems such as *ϵ* Aur, J1407, EE Cep and OGLE-LMC-ECL-11893 where the occultations are attributed to circumsecondary discs [18,25–27].

2 All normalized photometry is available at https://github.com/drgmk/rzpsc.

3 See http://wasp.cerit-sc.cz.

## Data accessibility

Materials used in the preparation of this contribution can be found online at https://github.com/drgmk/rzpsc.
